# Single-cell transcriptomic analysis reveals therapeutic mechanisms of adipose-derived stem cell exosomes in sepsis-induced lung injury

**DOI:** 10.1097/JS9.0000000000002894

**Published:** 2025-07-02

**Authors:** Shao-Chun Wu, Cheng-Shyuan Rau, Yi-Chan Wu, Chia-Wei Lin, Tsu-Hsiang Lu, Chia-Wen Tsai, Ming-Yu Yang, Ching-Hua Hsieh

**Affiliations:** a Department of Anesthesiology, Kaohsiung Chang Gung Memorial Hospital and Chang Gung University College of Medicine, Taiwan; b Graduate Institute of Clinical Medical Sciences, College of Medicine, Chang Gung University, Taoyuan, Taiwan; c Department of Neurosurgery, Kaohsiung Chang Gung Memorial Hospital and Chang Gung University College of Medicine, Taiwan; d Department of Plastic Surgery, Kaohsiung Chang Gung Memorial Hospital and Chang Gung University College of Medicine, Taiwan

**Keywords:** acute lung injury, adipose-derived stem cells, exosomes, sepsis, single-cell RNA sequencing

## Abstract

**Background:**

Sepsis-induced acute lung injury remains a leading cause of mortality in critically ill patients, with limited effective treatments beyond supportive care. This study investigates the therapeutic efficacy and underlying mechanisms of adipose-derived stem cell (ADSC) exosomes on sepsis-induced lung injury and characterizes underlying cellular and molecular mechanisms.

**Methods:**

We employed a cecal ligation and puncture (CLP) mouse model of sepsis and using male C57BL/6J mice (*Mus musculus*, 8–10 weeks old) and administered ADSC-derived exosomes intravenously. Animals were randomly assigned to Sham, CLP, or CLP + ADSC-exosome groups. Survival rates (*n* =12 for each group) and lung histopathology (*n* =5 for each group) were assessed. Single-cell RNA sequencing was performed on lung tissues to analyze cell type-specific transcriptomic changes and intercellular communication networks (*n* =2 for each group).

**Results:**

ADSC exosome treatment significantly improved survival rates and reduced lung pathology in CLP mice. Treatment altered lung cellular composition, increasing neutrophils, NKT cells, and monocytes while decreasing B and T cells. Gene expression analysis revealed downregulation of pro-inflammatory markers (TNF, IL-10, CCL3, and CCL4) and upregulation of tissue repair pathways. In neutrophils, exosomes reduced expression of respiratory burst genes while enhancing tissue repair mechanisms. In monocytes, treatment suppressed inflammatory cytokine production while promoting anti-inflammatory phenotypes. Exosome treatment is associated with transcriptomic changes suggestive of restored intercellular communication networks disrupted by sepsis, with increased signaling via CSF3, ANGPT, SPP1, and CCL pathways.

**Conclusion:**

ADSC-derived exosomes effectively treat sepsis-induced lung injury by rebalancing the cellular environment and restoring homeostasis through modulation of immune cell function and intercellular communication, offering potential as a cell-free novel therapeutic approach for sepsis-related pulmonary complications.

## Introduction

Sepsis is defined as “life-threatening organ dysfunction caused by a dysregulated host response to infection,” according to the Third International Consensus (Sepsis-3)^[1]^. Sepsis is typically initiated when multiple infection-related microbial products and endogenous danger signals are simultaneously detected by complement proteins and specific cell surface receptors. The activation of these various signaling pathways leads to the expression of several key gene families^[2]^. A large-scale release of proinflammatory cytokines and reactive oxygen species (ROS) by macrophages and neutrophils, which disrupts vascular permeability, impairs cardiac function, and triggers metabolic changes, ultimately leading to tissue necrosis and organ failure^[3]^. The proinflammatory response is followed by an anti-inflammatory phase that results in functional immunosuppression.

Patients with sepsis are at heightened risk for acute respiratory failure^[4,5]^. Approximately 50% of patients with severe sepsis will go on to develop acute lung injury (ALI) or acute respiratory distress syndrome (ARDS)^[6]^. Neutrophils are thought to play a central role in the development of ALI and ARDS, with their activation and transmigration being key events in the progression of these conditions^[7]^. Excessive activation of neutrophils causes tissue damage through the release of cytotoxic and immune-stimulating agents, including proteinases, cationic polypeptides, cytokines, and ROS^[7,8]^. Macrophages also play dual roles in sepsis-induced ALI. In the early phase of sepsis, pro-inflammatory signals like interferon-γ (IFN-γ) and lipopolysaccharide (LPS) trigger the polarization of macrophages toward an M1-like state. These M1-like macrophages proliferate and secrete large amounts of inflammatory mediators, including interleukin-1 (IL-1), tumor necrosis factor-α (TNF-α), interleukin-6 (IL-6), and ROS, leading to intense inflammatory responses. In contrast, during the late stage of sepsis, an excessive accumulation of M2-like macrophages results in the production of anti-inflammatory cytokines such as interleukin-10 (IL-10) and transforming growth factor-β (TGF-β), driving the host into an immunosuppressive state^[9,10]^. In sepsis, the adaptive immune response also becomes dysregulated or impaired, resulting in inadequate infection control and/or immune suppression. Adaptive immune cells undergo significant cell death or T cell exhaustion. CD4^+^ T cells are particularly vulnerable, exhibiting high rates of apoptosis and impaired secretory functions. Regulatory T cells tend to resist apoptosis, playing a key role in driving the immune suppression characteristic of sepsis^[11]^.

Despite tremendous advances in understanding the origin, pathophysiology and immunological mechanisms of sepsis, it remains one of the leading causes of morbidity and mortality in critically sick patients^[12]^. Mortality has only been demonstrated to be decreased by prompt fluid resuscitation and early broad-spectrum antibiotic treatment^[13]^. The timing of the accurate diagnosis and the start of the supportive, adjunctive, and causative interventions are critical factors. The cecal-ligation-and-puncture (CLP) mouse model remains the benchmark pre-clinical platform because it produces a polymicrobial infection and pathophysiologic cascade – early hyper-inflammation, hemodynamic collapse, and late immunosuppression – that mirror the Sepsis-3 phenotype and its downstream organ injuries, notably ALI^[3]^. Comparative transcriptomic and physiologic analyses show a high concordance between CLP and human sepsis signatures, while therapeutic signals that improve CLP survival frequently predict benefit in large-animal studies and early-phase trials^[14,15]^. Consequently, CLP offers strong construct and predictive validity for probing mechanisms and evaluating candidate interventions in human sepsis.

Adipose-derived stem cell (ADSC)-derived exosomes play a crucial role in intercellular communication by transferring bioactive molecules, such as proteins, lipids, and nucleic acids, to target cells^[16]^. They have attracted significant attention in regenerative medicine and therapeutic applications due to their versatile functions^[17,18]^. ADSC-derived exosomes have been shown to reduce sepsis-induced lung injury in mouse model^[19,20]^. In this study, we utilized single-cell RNA sequencing (scRNA-seq) to comprehensively investigate the cellular responses and transcriptomic heterogeneity among various lung cell types in a CLP mouse model, both during the sepsis and following treatment with ADSC-derived exosomes.

## Materials and methods

### ADSC culture conditions

Mouse ADSCs (100MU006, iXCells biotechnologies) were cultured in ADSC growth medium (DM-0003, iXCells biotechnologies) supplemented with 100 units/ml penicillin and streptomycin at 37°C under a humidified atmosphere of 95% air, 5% CO_2,_ and passaged at 80–90% confluence using 0.25% trypsin-EDTA. ADSCs from passages 3–5 were used for subsequent experiments to ensure optimal secretory profiles. Cells were authenticated by flow cytometry for stem cell markers (CD29+, CD44+, CD73+, CD90+, CD105+) and absence of hematopoietic markers (CD34-, CD45-).

**HIGHLIGHTS**

- ADSC-derived exosomes improve survival in CLP-induced sepsis models
- Single-cell sequencing reveals immune cell composition rebalancing
- Key inflammatory mediators (TNF, IL-10, CCL3) are downregulated
- Tissue repair pathways are enhanced after exosome treatment
- Intercellular communication networks disrupted by sepsis are restored
- Potential therapeutic approach for sepsis-induced lung injury

### Exosome purification and characterization

ADSC-derived exosomes were isolated using the ExoQuick-TC™ exosome precipitation solution (EXOTC50A-1, System Biosciences) according to the manufacturer’s protocol with modifications. Briefly, ADSCs were cultured to 80% confluence in complete medium, then switched to serum-free medium for 48 h to collect conditioned media containing secreted exosomes. The conditioned media were centrifuged sequentially at 300 × *g* for 10 min and 3000 × *g* for 15 min to remove cells and cellular debris. The supernatant was filtered through a 0.22 μm filter and mixed with ExoQuick-TC solution at a 5:1 ratio (media). The mixture was refrigerated at 4°C overnight (12–16 h), followed by centrifugation at 1500 × *g* for 30 min. The resulting exosome pellet was resuspended in sterile PBS and quantified using the BCA protein assay (23 225, Thermo Scientific). Exosome characterization was performed using transmission electron microscopy, nanoparticle tracking analysis (size distribution: 50–150 nm), and Western blotting for exosome markers (CD9, CD63, CD81, TSG101) according to our previous works^[21–24]^.

### Animal housing and ethical considerations

Male C57BL/6 J mice (*Mus musculus*, 8–10 weeks old, weighing 25–30 g) were purchased from the National Laboratory Animal Center, NARLabs, Taiwan. Animals were housed in a specific pathogen-free facility under controlled conditions (12-h light/dark cycle, 22 ± 2°C, 50 ± 10% humidity) with ad libitum access to standard laboratory chow and water. All animals were acclimatized for at least 7 days before experimentation. The study protocol was approved by the Institutional Animal Care and Use Committee and conducted in accordance with the Guide for the Care and Use of Laboratory Animals published by the US National Institutes of Health. The facility was accredited by the Association for Assessment and Accreditation of Laboratory Animal Care International. The work has been reported in accordance with the ARRIVE guidelines (Animals in Research: Reporting In Vivo Experiments)^[25]^.

### Cecal ligation and puncture sepsis model

The mice were randomly divided into three groups: control, CLP, and exosome. Survival rates (*n* =12 per group), lung histopathology (*n* =5 per group), and scRNA-seq (*n* =2 per group) were evaluated. Sepsis was established through CLP as previously described^[3]^. Surgery is performed through midline abdominal incision. The cecum was then ligated below the ileocecal valve and punctured once with a 21-gauge needle before being compressed to extract a fraction of the feces. Following surgery, the CLP mice were resuscitated with a subcutaneous injection of 5 mL of pre-warmed normal saline per 100 g body weight. The control group consisted of mice that only received cecum exposure without conducting CLP. In the exosome group, a total of two injections of 100 μg of ADSC-derived exosomes were administered via the tail vein at 4-h intervals after surgery. Mice were monitored every 12 h for survival analysis over 10 days, while a separate cohort was sacrificed at 12 h post-surgery for tissue collection and subsequent analyses. The animals’ respiratory rate and body temperature were monitored daily from the postoperative period until death or 10 days later.

### Tissue harvest and single-cell preparation

Both mouse lung samples were processed using the lung dissociation kit (Miltenyi Biotec, 130-095-927). Briefly, lungs were dissected into single lobes and washed in a petri plate with PBS (pH 7.2) to eliminate any leftover blood. The lobes were transferred to a new tube containing the enzyme and incubated for 30 min at 37°C with constant rotation. The resulting cell suspension was filtered through a 70 μm MACS SmartStrainer and washed with DMEM containing 10% FBS. Red blood cells were lysed using Red Blood Cell Lysis Solution (130-094-183, Miltenyi Biotec) for 10 min at room temperature. Debris was removed using Debris Removal Solution (130-109-398, Miltenyi Biotec) followed by centrifugation at 300 × *g* for 10 min. Dead cells were eliminated using the Dead Cell Removal Kit (130-090-101, Miltenyi Biotec) according to the manufacturer’s protocol. Cell viability and concentration were assessed using trypan blue exclusion and a hemocytometer, with only preparations showing >85% viability proceeding to scRNA-seq.

### 10X single-cell library construction and sequencing

The mouse lung cells were processed for scRNA-seq using the GemCode Single Cell Platform. Libraries were prepared using the Chromium Next GEM Single Cell 3ʹ Kit v3.1 (10X Genomics, Pleasanton), according to the manufacturer’s protocols. Briefly, cell suspensions were loaded on a Chromium Single-Cell Chip G along with the reverse transcription master mix and single cell 3ʹ gel beads. Cells were lysed in individual droplets, and the mRNA was linked to the cellular barcodes on the beads to form a single-cell gel bead-in-emulsions. A reverse transcription reaction was performed in droplets to construct a cDNA library by Applied Biosystems™ Veriti™ 96-Well Thermal Cycler (Thermo Fisher Scientific, USA). Amplified cDNA was purified with SPRIselect beads and converted to the sequencing library. These sample libraries were then sequenced using an Illumina Novaseq System (Illumina, Inc., San Diego, CA, USA) with 150-bp paired-end reads.

### Computational analysis of single-cell transcriptomes

Raw sequencing data were processed using the Cell Ranger pipeline (v6.1.1, 10X Genomics) to perform quality control, barcode processing, and UMI counting. FASTQ files were aligned to the mouse reference genome (GRCm38/mm10) using STAR aligner incorporated in Cell Ranger. The filtered gene-barcode matrices were then analyzed using the Seurat package (v4.0.5) in R (v4.1.0). During quality control, cells with less than 200 identified genes or more than 20% mitochondrial content were filtered out to exclude low-quality cells and potential doublets. After quality control, each dataset was normalized using SCTransform, and the top 2000 highly variable genes were identified using the “FindVariableFeatures” function with “vst” method. Batch effects between samples were mitigated using the “IntegrateData” function. Principal component analysis was performed on the variable genes, with the first 30 principal components used for downstream analyses. Uniform Manifold Approximation and Projection (UMAP) dimensionality reduction was applied for visualization. Cell clusters were identified using the “FindNeighbors” and “FindClusters” functions with a resolution parameter of 0.5. Cell type annotation was performed using the SingleR package (v1.6.1) with reference to the ImmGen database, followed by manual curation based on canonical marker genes.

### Differential gene expression analysis

Differential gene expression analysis between experimental groups (Sham vs. CLP and CLP vs. EXO) for each identified cell type was conducted using the “FindMarkers” function in Seurat, employing the Wilcoxon rank-sum test. DEGs were identified using stringent criteria: |log2 fold change| > 2 and adjusted *p*-value < 0.05 after Benjamini–Hochberg correction for multiple testing. For each cell type, volcano plots were generated to visualize the distribution of DEGs, with upregulated genes shown in red and downregulated genes in green.

### Pathway and functional enrichment analysis

Functional enrichment analyses were performed using the clusterProfiler R package (version 3.18.1). For each set of DEGs (upregulated and downregulated separately), GO enrichment analysis was conducted to identify significantly overrepresented biological process (BP) terms. KEGG pathway enrichment analysis was performed to identify significantly enriched molecular pathways. Enrichment significance was determined using a hypergeometric test with Benjamini–Hochberg correction for multiple testing. Terms with adjusted *p*-value < 0.05 were considered significant. Results were visualized using heatmaps for GO terms and bar plots for KEGG pathways, with color intensity representing statistical significance. To identify relevant literature, a comprehensive search was conducted across PubMed, Scopus, and Web of Science databases. The search covered publications from January 2015 to March 2025. Combinations of the following keywords were used: “adipose-derived stem cell exosomes,” “ADSC exosomes,” “sepsis,” “acute lung injury,” “single-cell RNA sequencing,” and “scRNA-seq.” Boolean operators (AND, OR) were applied to refine results, and filters were set for original research articles, reviews, and English language only. Articles were screened for relevance based on title, abstract, and full text, focusing on those reporting preclinical or mechanistic findings related to exosome therapy in sepsis models.

## Results

### ADSC-derived exosomes rescue mice from sepsis-induced mortality and pulmonary damage

**Figure 1.**
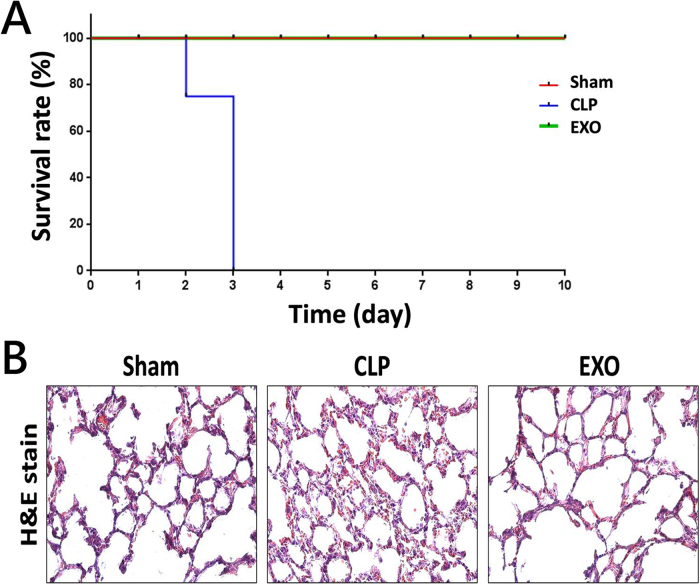
ADSC-derived exosomes treatment alleviated the lung injury of CLP-induced sepsis. (A) Kaplan–Meier survival curve showing the survival rates of mice across three experimental groups (Sham, CLP, and EXO) over a 10-day observation period (*n* =12 per group). Note the 100% mortality by day 3 in the untreated CLP group compared to 100% survival in both Sham and EXO groups, highlighting the protective efficacy of ADSC-derived exosome treatment. (B) Representative hematoxylin and eosin (H&E) staining images (200× magnification) of lung tissue sections from each experimental group. The Sham group shows normal lung architecture with thin alveolar walls and open-air spaces. The CLP group exhibits severe lung injury characterized by increased septal thickening, inflammatory cell infiltration, and alveolar collapse. In contrast, the EXO group shows markedly improved lung histology with reduced inflammatory infiltration and preserved alveolar structure, indicating the therapeutic effect of ADSC-derived exosomes in mitigating sepsis-induced lung damage.

We primarily monitored the survival of CLP-induced mice, with or without ADSC-derived exosome treatment, over a 10-day period to evaluate the therapeutic effects of ADSC-derived exosomes on sepsis-induced mortality. The results indicated that CLP mice exhibited a 75% mortality rate within 2 days and reached 100% mortality by day 3 (Fig. 1A). Treatment with ADSC-derived exosomes significantly improved the survival of mice compared to untreated CLP mice, with all treated mice surviving throughout the 10-day observation period. The lung tissue condition of the mice was assessed using hematoxylin and eosin (H&E) staining. The findings revealed that CLP mice exhibited signs of lung injury (Fig. 1B). However, this CLP-induced lung injury was mitigated by treatment with ADSC-derived exosomes. Therefore, these results indicated that the overall survival and CLP-induced lung injury were alleviated after ADSC-derived exosomes treatment.

### Comprehensive single-cell profiling reveals dynamic shifts in lung cellular composition

**Figure 2.**
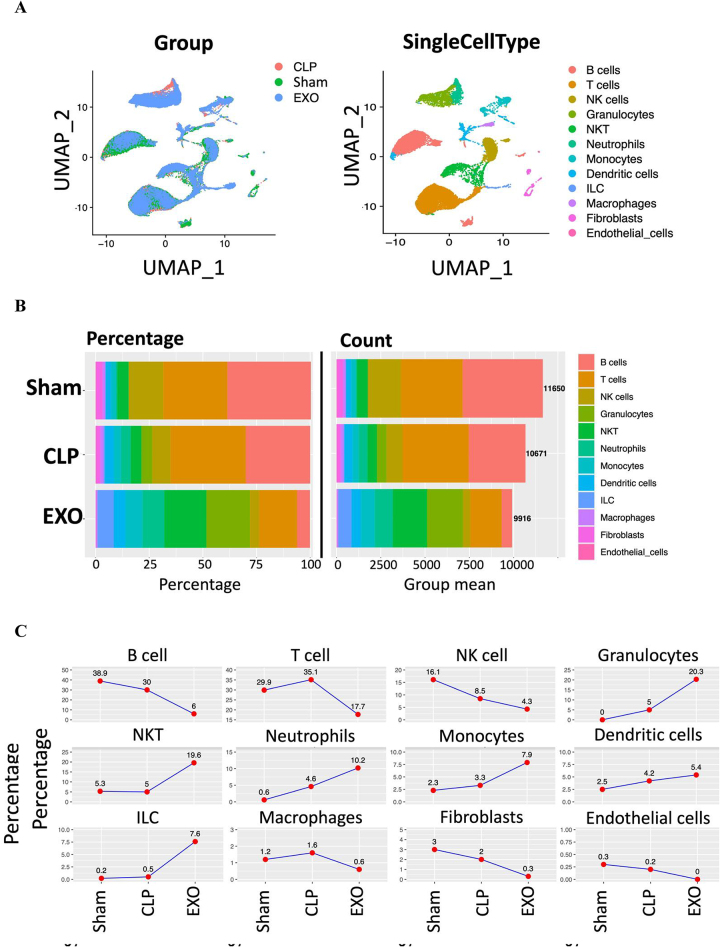
Single-cell analyses reveal the cellular composition of the mouse lung following sepsis induction and treatment with ADSC-derived exosomes. (A) UMAP visualization of unsupervised scRNA-seq clustering analysis identifying 12 distinct cellular populations in lung tissue, with each cell type designated by a unique color. The plot illustrates the transcriptional relationships between different cell types, revealing clear clustering patterns based on cell identity. (B) Comprehensive bar charts depicting both the relative proportion (percentage, left panel) and absolute counts (right panel) of each identified cell type within lung samples from Sham, CLP, and EXO groups. Numbers on the right side indicate total cell counts per group. Note the significant shifts in cellular composition across experimental conditions. (C) Detailed comparison of the relative abundance of each cell type across experimental groups, presented as line graphs with statistical values. Note the significant decrease in B cells, T cells, and NK cells in the EXO group compared to Sham and CLP, while granulocytes, NKT cells, neutrophils, monocytes, and ILCs show marked increases after ADSC-exosome treatment, highlighting exosome therapy’s immunomodulatory effects in the septic lung.

To investigate the lung injury effects of CLP-induced mice, with or without treatment using ADSC-derived exosomes, we isolated cells from lung tissue samples and performed scRNA-seq using the 10× Genomics platform. Each group included two biological replicates, enabling us to analyze the cellular and transcriptomic alterations in the lung microenvironment following sepsis induction and subsequent exosome treatment. After standard data processing, the UMAP analysis revealed the scRNA-seq data for lung tissue (Fig. 2A). Totally, we clustered and annotated these cells into 12 cell types, including B cells, T cells, NK cells, granulocyte, NKT cells, neutrophil, monocyte, dendritic cells, innate lymphoid cells (ILC), macrophages, fibroblasts, and endothelial cells.

To reveal the cellular composition of lung tissue across groups, we analyzed and compared the relative percentages of each cell type. The percentages and absolute counts of all cell types within each group were shown in Figure 2B. The changes in the relative abundance of each cell type between groups were shown in Figure 2C. In the sham and CLP groups, B cells, T cells, and NK cells were the three most prevalent subsets. However, treatment with ADSC-derived exosomes significantly altered the cellular composition, with granulocytes, NKT cells, and T cells becoming the dominant subsets. Notably, treatment with ADSC-derived exosomes led to a significant decrease in the percentages of B cells, T cells, and NK cells, while the proportions of granulocytes, NKT cells, neutrophils, monocytes, and ILC markedly increased (Fig. 2C). These findings suggested that ADSC-derived exosomes may exert their regulatory effects on the septic lung of these immune cells. Next, we further evaluated the transcriptomic features of each cell type.

### Sepsis and exosome treatment induce distinct transcriptional signatures

**Figure 3.**
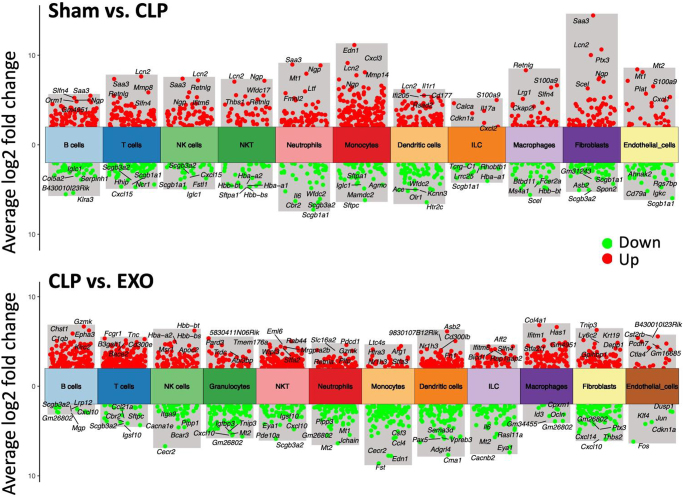
Differential gene expression analysis of lung cellular populations across experimental groups. Comprehensive volcano plots illustrating differentially expressed genes (DEGs) in each cell type for two key comparisons: Sham vs. CLP (top panels) and CLP vs. EXO (bottom panels). Each dot represents an individual gene, with red dots indicating significantly upregulated genes (log2FC > 2, *q* < 0.05) and green dots indicating significantly downregulated genes (log2FC < − 2, *q* < 0.05). The *x*-axis shows log2 fold change, while the *y*-axis represents statistical significance as -log10. Note the distinct gene expression patterns across different cell types, reflecting cell-specific responses to sepsis and subsequent exosome treatment. These plots provide a global view of transcriptional changes and highlight key regulatory genes involved in the pathogenesis of sepsis-induced lung injury and its resolution following ADSC-exosome treatment.

We performed differential gene expression analysis comparing Sham versus CLP and CLP versus EXO groups across all lung cell populations (Fig. 3). Neutrophils exhibited 52 DEGs in Sham versus CLP comparison (43 upregulated, 9 downregulated), with increased expression of activation markers (CD177, Ly6G) and inflammatory mediators (Cxcl3, IL-10). Monocytes showed 66 DEGs (53 upregulated, 13 downregulated), including elevated TNF and chemokines (CCL3, CCL4). Fibroblasts displayed 52 DEGs (24 upregulated, 28 downregulated), with increased expression of inflammatory cytokines (IL6, CXCL1).

ADSC-exosome treatment reversed many sepsis-induced transcriptional changes, with 41 DEGs in neutrophils, 37 in monocytes, and 93 in fibroblasts when comparing CLP versus EXO groups. Key inflammatory genes (IL-10, TNF, CCL3) were significantly downregulated, while tissue repair factors were upregulated. Intersection analysis revealed a core set of genes modulated by sepsis and subsequently normalized by exosome treatment (Table 1), demonstrating targeted reversal of sepsis-induced transcriptional dysregulation.

**Table 1:** Genes impacted by CLP that were restored after treatment with ADSC-derived exosomes

Genes	Cell type	Sham vs. CLP		CLP vs. EXO	
		Log2fc	*p* value	Log2fc	*p* value
Bank1	B cells	−2.426	0.000	2.523	0.000
Gm31243	B cells	−2.539	0.000	2.427	0.000
Iglc1	B cells	−3.402	0.000	4.654	0.000
Plcb1	Dendritic cells	−3.171	0.000	2.173	0.000
Tppp3	Dendritic cells	−2.600	0.000	2.245	0.000
Adm	Fibroblasts	2.471	0.000	−3.183	0.000
Bmp6	Fibroblasts	−2.076	0.037	2.681	0.031
Cxcl1	Fibroblasts	3.473	0.000	−3.933	0.002
Fst	Fibroblasts	2.075	0.000	−3.093	0.034
Il6	Fibroblasts	2.650	0.000	−3.175	0.002
Kcne4	Fibroblasts	2.206	0.000	−2.832	0.000
Myct1	Fibroblasts	−2.268	0.014	4.215	0.000
Ntrk2	Fibroblasts	2.150	0.000	−2.108	0.015
Rhbdd2	Fibroblasts	2.653	0.000	−3.434	0.010
Tcrg-C1	ILC	−2.963	0.000	2.638	0.000
Ccl3	Monocytes	4.121	0.000	−4.116	0.000
Ccl4	Monocytes	6.220	0.000	−5.803	0.000
Cxcl3	Monocytes	9.134	0.000	−3.323	0.000
Edn1	Monocytes	11.138	0.000	−7.748	0.000
Fnip2	Monocytes	3.282	0.000	−2.101	0.000
Hfe	Monocytes	−2.306	0.000	2.033	0.000
Ifi205	Monocytes	3.828	0.000	−2.398	0.000
Il1rn	Monocytes	5.209	0.000	−2.641	0.000
Lst1	Monocytes	−2.084	0.000	2.198	0.000
Met	Monocytes	3.530	0.000	−2.199	0.000
Mt1	Monocytes	6.295	0.000	−2.507	0.000
Nrg1	Monocytes	5.870	0.000	−2.527	0.000
Oasl1	Monocytes	4.823	0.000	−3.650	0.000
Rsad2	Monocytes	4.035	0.000	−2.889	0.000
Tnf	Monocytes	2.010	0.000	−2.860	0.000
Tnip3	Monocytes	2.141	0.000	−2.646	0.000
F10	Neutrophils	4.791	0.000	−2.676	0.000
Fabp5	Neutrophils	−2.230	0.000	2.925	0.000
Fnip2	Neutrophils	3.552	0.000	−2.606	0.000
Hmox1	Neutrophils	3.470	0.000	−2.304	0.000
Il10	Neutrophils	4.452	0.000	−3.752	0.000
Mreg	Neutrophils	3.635	0.000	−3.042	0.000
Mt1	Neutrophils	7.939	0.000	−4.789	0.000
Pi16	Neutrophils	−2.054	0.000	2.093	0.000
Saa3	Neutrophils	8.959	0.000	−3.733	0.000
Rap1gap2	NK cells	−2.492	0.000	2.061	0.000
Gzmk	NKT	−2.102	0.000	3.986	0.000

### Neutrophil phenotype transitions from pro-inflammatory to reparative following exosome therapy

**Figure 4.**
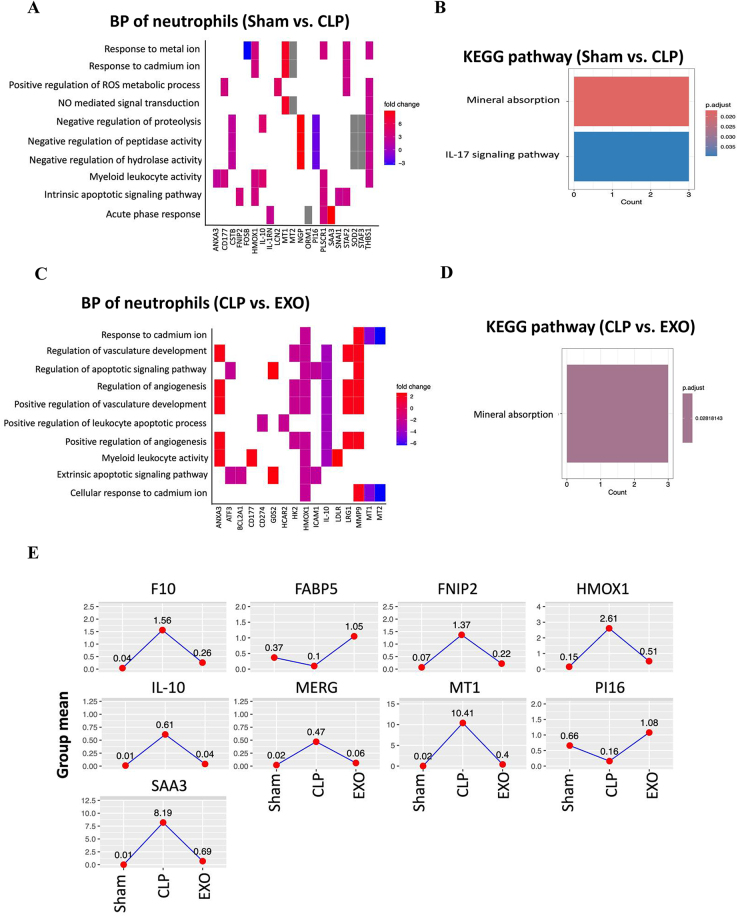
Functional enrichment analysis of differentially expressed genes in neutrophils. (A) Heatmap showing biological process (BP) enrichment of DEGs in neutrophils from Sham vs. CLP comparison, highlighting pathways related to metal ion response, ROS metabolism, and acute phase response. (B) KEGG pathway enrichment for neutrophil DEGs in Sham vs. CLP comparison, showing significant enrichment in mineral absorption and IL-17 signaling. (C) Heatmap of BP enrichment for neutrophil DEGs in CLP vs. EXO comparison, with emphasis on cadmium ion response, vasculature development, and leukocyte apoptotic regulation. (D) KEGG pathway enrichment for neutrophil DEGs in CLP vs. EXO comparison. (E) Expression profiles of key neutrophil genes (F10, FABP5, FNIP2, HMOX1, IL-10, MREG, MT1, PI16, SAA3) across experimental groups, showing induction by sepsis and normalization following exosome treatment.

The primary pathological characteristic of septic lung injury is the excessive accumulation and activation of neutrophils within lung tissue. We observed a significant increase in neutrophil composition in the CLP group compared to the Sham group (4.6 ± 2.3% vs. 0.6 ± 0.4%, *p* < 0.001) (Fig. 2C). The full list of DEGs per cell type in the comparison of CLP vs. Sham groups is provided in the Supplemental Digital Content Table 1, available at, http://links.lww.com/JS9/E555. A total of 52 DEGs (43 upregulated and 9 downregulated) were detected. *CD177*, a specific marker of neutrophil activation, was markedly elevated in the CLP group. *Ly6G*, a homolog of the human *CD177* known to interact with cell adhesion molecules, was also significantly elevated in the CLP group. Additionally, the gene expression levels of several cytokines and chemokines, such as *Cxcl3, IL-10, IL1RN*, and *IL1F9*, were significantly upregulated. The gene ontology analysis showed that DEGs were mainly involved in response to metal ion, ROS and NO process, myeloid leukocyte activation, and acute phase response (Fig. 4A). The KEGG pathway enrichment analysis showed these DEGs involved in mineral absorption and IL-17 signaling pathway (Fig. 4B).

Following treatment with ADSC-derived exosomes, the proportion of neutrophils significantly increased to 10.2 ± 5.7%, which is significantly higher than those in the CLP group and in the Sham group (both *p* < 0.001 in Chi-square test) (Fig. 2C). The full list of DEGs per cell type in the comparison of ADSC-derived exosomes vs. CLP groups is provided in the Supplemental Digital Content Table 2, available at, http://links.lww.com/JS9/E556. A total of 41 DEGs (20 upregulated and 21 downregulated) were detected (Supplemental Digital Content Table 2, available at, http://links.lww.com/JS9/E556). We observed a continuous increase in the expression level of *CD177. Matrix metalloprotease 9 (MMP-9)* and *MMP-25* were significantly upregulated in EXO group. Notably, *IL-10, CCRL2*, and *CD274* (also known as *PD-L1*) were significantly reduced following treatment with ADSC-derived exosomes. The gene ontology analysis showed that DEGs were mainly involved in response to cadmium ion, regulation of vasculature development and angiogenesis, regulation of leukocyte apoptotic process and myeloid leukocyte activity (Fig. 4C). The KEGG pathway enrichment analysis showed these DEGs involved in mineral absorption (Fig. 4D).

From the intersection of these two DEGs sets, we identified genes regulated by CLP that were restored following treatment with ADSC-derived exosomes (Fig. 4E). Among these, *F10, FNIP2, HMOX1, IL-10, MERG, MT1*, and *SAA3* exhibited increased expression following CLP, which was subsequently reduced after treatment with ADSC-derived exosomes. In contrast, *FABP5* and *PI16* showed decreased expression after CLP, which was later increased by treatment with ADSC-derived exosomes.

### Exosomes reprogram monocyte function towards anti-inflammatory phenotypes

**Figure 5.**
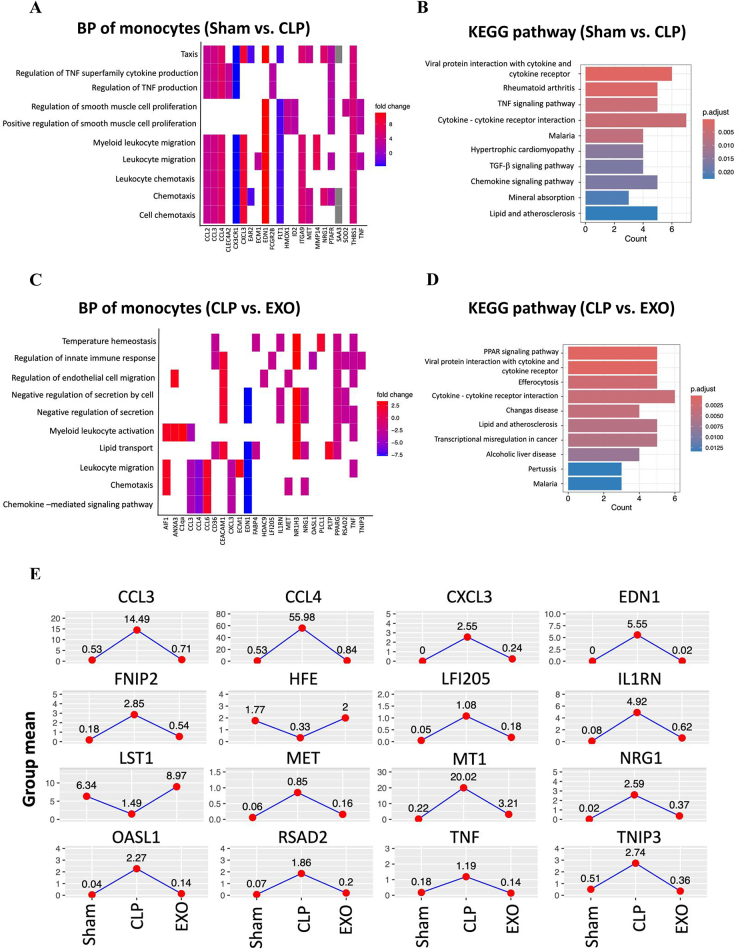
Functional enrichment analysis of differentially expressed genes in monocytes. (A) Heatmap of BP enrichment for monocyte DEGs in Sham vs. CLP comparison, showing enrichment in chemotaxis, TNF regulation, and leukocyte migration pathways. (B) KEGG pathway enrichment for monocyte DEGs in Sham vs. CLP comparison. (C) Heatmap of BP enrichment for monocyte DEGs in CLP vs. EXO comparison, focusing on immune response regulation, endothelial cell migration, and secretion pathways. (D) KEGG pathway enrichment for monocyte DEGs in CLP vs. EXO comparison. Efferocytosis is the process by which certain immune cells, like macrophages, recognize, engulf, and remove dead or dying cells from the body. This cleanup helps maintain tissue health and prevents inflammation caused by cell debris. (E) Expression profiles of key monocyte genes across experimental groups, demonstrating pro-inflammatory gene induction during sepsis and subsequent normalization after exosome treatment.

During the initial stages of sepsis, monocytes become activated and exhibit both functional and morphological changes in response to infectious stimuli. We noted a modest increase in monocyte composition in the CLP group compared to the Sham group (3.3 ± 0.5%% vs. 2.3 ± 0.2%, respectively, *p* = 0.14) (Fig. 2C). A total of 66 DEGs (53 upregulated and 13 downregulated) were detected (Supplemental Digital Content Table 1, available at, http://links.lww.com/JS9/E555). Tumor necrosis factor (TNF) was markedly elevated in the CLP group. The expression levels of several cytokine and chemokine genes, including *CCL2, CCL3, CCL4, IL1RN,* and *CXCL3,* were also significantly increased. The gene ontology analysis revealed that DEGs were primarily associated with processes such as response to chemotaxis, regulation of TNF and related cytokine production, regulation of smooth muscle cell proliferation, and leukocyte chemotaxis and migration (Fig. 5A). The KEGG pathway enrichment analysis revealed that these DEGs are associated with cytokine–cytokine receptor interaction, TNF signaling pathway, TGF-β signaling pathway, and chemokine signaling pathway (Fig. 5B).

Following treatment with ADSC-derived exosomes, the proportion of monocytes significantly increased to 7.9% (Fig. 2C). A total of 37 DEGs (15 upregulated and 22 downregulated) were detected (Supplemental Digital Content Table 2, available at, http://links.lww.com/JS9/E556). *CCL6* levels were markedly elevated in the EXO group. Interestingly, *TNF, CCL3, CCL4, IL1RN*, and *CXCL3* were significantly decreased after treatment with ADSC-derived exosomes. Similarly, *CD36* and *CD83* were also significantly reduced following the treatment. The gene ontology analysis showed that DEGs were mainly involved in regulation of innate immune response and endothelial cell migration, negative regulation of secretion, leukocyte activation and migration, chemotaxis and chemokine – mediated signaling pathway (Fig. 5C). The KEGG pathway enrichment analysis showed these DEGs involved in PPAR signaling pathway, cytokine–cytokine receptor interaction and efferocytosis, a process by which phagocytic cells recognize, engulf, and digest apoptotic cells, clearing them efficiently and silently to maintain tissue homeostasis and prevent inflammation (Fig. 5D).

From the intersection of these two DEGs sets, we found *CCL3, CCL4, CXCL3, EDN1, FNIP2, LFI205, IL1RN, MET, MT1, NRG1, OASL1, RSAD2, TNF,* and *TNIP3* exhibited increased expression following CLP, which was subsequently reduced after treatment with ADSC-derived exosomes. In contrast, *HFE* and *LST1* showed decreased expression after CLP, which was later increased by treatment with ADSC-derived exosomes.

### Resident fibroblasts demonstrate reduced pro-fibrotic activation after exosome treatment

**Figure 6.**
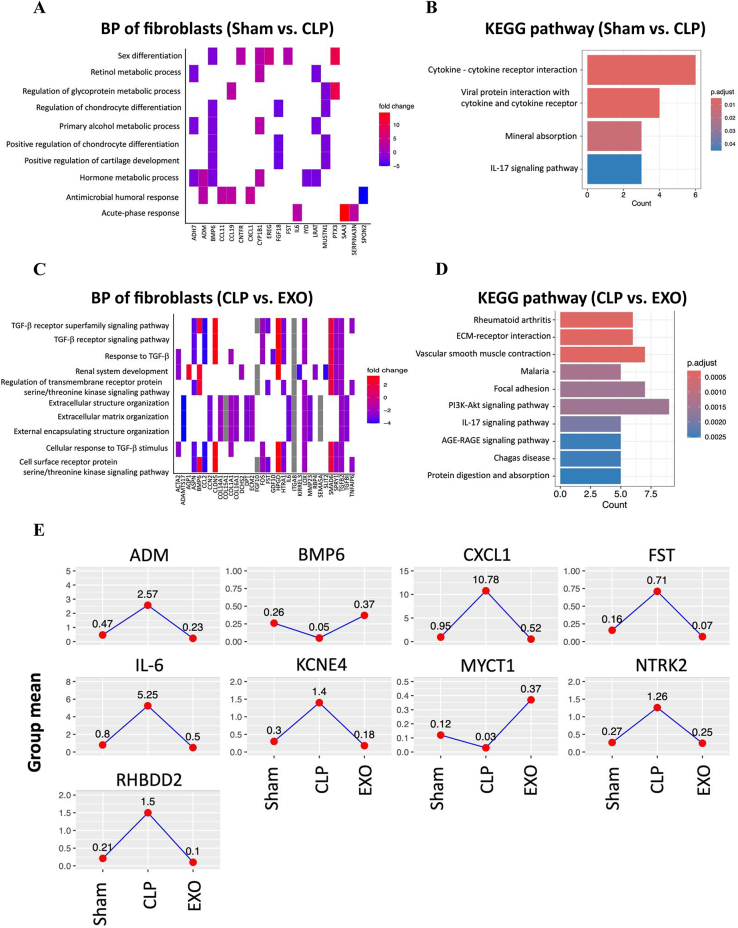
Functional enrichment analysis of differentially expressed genes in fibroblasts. (A) Heatmap of BP enrichment for fibroblast DEGs in Sham vs. CLP comparison, highlighting sex differentiation, metabolic processes, and acute-phase response. (B) KEGG pathway enrichment for fibroblast DEGs in Sham vs. CLP comparison. (C) Heatmap of BP enrichment for fibroblast DEGs in CLP vs. EXO comparison, with emphasis on TGF-β signaling and extracellular matrix organization. (D) KEGG pathway enrichment for fibroblast DEGs in CLP vs. EXO comparison. The AGE-RAGE pathway involves advanced glycation end-products (AGEs), which are harmful molecules formed when sugars attach to proteins or fats without enzymes. When AGEs activate RAGE, it triggers harmful effects inside cells, including inflammation and oxidative stress. (E) Expression profiles of key fibroblast genes (ADM, BMP6, CXCL1, FST, IL-6, KCNE4, MYCT1, NTRK2, RHBDD2) across experimental groups, showing pro-inflammatory and pro-fibrotic gene induction during sepsis and normalization after exosome treatment.

Activated and inactivated subsets of fibroblasts have been associated with inflammatory responses by affecting the proliferation, migration, retention, and apoptosis of infiltrating immune cells^[26]^. We noted a modest decrease in fibroblasts composition in the CLP group compared to the Sham group (3.0 ± 0.4% vs. 2.0 ± 0.1%, respectively, *p* = 0.14) (Fig. 2C). A total of 52 DEGs (24 upregulated and 28 downregulated) were detected (Supplemental Digital Content Table 1, available at, http://links.lww.com/JS9/E555). The expression levels of several cytokine and chemokine genes, such as IL6, CCL11, CCL19, and CXCL1, showed a significant increase. The gene ontology analysis showed that DEGs were mainly involved in antimicrobial humoral response and acute-phase response (Fig. 6A). The KEGG pathway enrichment analysis showed these DEGs involved in cytokine-cytokine receptor interaction and IL-17 signaling pathway (Fig. 6B).

Following treatment with ADSC-derived exosomes, the proportion of fibroblasts significantly increased to 3% (Fig. 2C). A total of 93 DEGs (37 upregulated and 56 downregulated) were detected (Supplemental Digital Content Table 2, available at, http://links.lww.com/JS9/E556). The expression levels of various cytokine and chemokine genes, including IL6, CCL2, CXCL1, and CXCL14, were significantly decreased. Similarly, TGFB2, TGFBI, and GDF10 exhibited a notable reduction in the CLP group. In contrast, the levels of CD36 and CD93 were markedly increased in the EXO group, along with a substantial elevation in EGFL7 and FGF10. The gene ontology analysis showed that DEGs were mainly involved in TGF-β signaling pathway, serine/threonine kinase signaling pathway, and extracellular matrix organization (Fig. 6C). The KEGG pathway enrichment analysis showed these DEGs involved in ECM-receptor interaction, focal adhesion, PI3K-Akt signaling pathway, IL-17 signaling pathway, and AGE-RAGE signaling pathway (Fig. 6D). The IL-17 signaling pathway begins with the binding of IL-17A/F to IL-17RA/RC receptors, then triggers NF-κB, MAPK, and C/EBP pathways and induces proinflammatory gene expression and stabilizing mRNAs to promote inflammation^[27]^. The AGE-RAGE pathway involves advanced glycation end-products binding to their receptor RAGE, triggering intracellular signaling cascades, such as NF-κB, STAT3, and MAPKs. This activation promotes inflammation, oxidative stress, apoptosis, and vascular dysfunction^[28]^.

From the intersection of these two DEGs sets, we found *CCL3, CCL4, CXCL3, EDN1, FNIP2, LFI205, IL1RN, MET, MT1, NRG1, OASL1, RSAD2, TNF,* and *TNIP3* exhibited increased expression following CLP, which was subsequently reduced after treatment with ADSC-derived exosomes (Fig. 6E). In contrast, *HFE* and *LST1* showed decreased expression after CLP, which was later increased by treatment with ADSC-derived exosomes.

Fibroblasts play crucial roles in inflammatory responses by influencing immune cell behavior and mediating tissue repair or fibrosis. In our CLP model, fibroblast population decreased modestly, with significant transcriptional reprogramming comprising 52 DEGs (24 upregulated, 28 downregulated). Key proinflammatory and profibrotic genes including IL6, CCL11, CCL19, and CXCL1 were markedly upregulated in septic lungs. Gene ontology analysis revealed enrichment in antimicrobial response and acute-phase pathways (Fig. 6A), with KEGG analysis highlighting cytokine–receptor interactions and IL-17 signaling (Fig. 6B). Following exosome treatment, fibroblast percentage increased to 0.3%, with substantial transcriptional changes (93 DEGs: 37 upregulated, 56 downregulated). Notably, proinflammatory cytokines (IL6, CCL2, CXCL1) and TGF-β pathway components (TGFB2, TGFBI, GDF10) were significantly downregulated, while tissue repair factors (CD36, CD93, EGFL7, FGF10) were upregulated. Functional analysis showed enrichment in TGF-β signaling regulation and ECM organization (Fig. 6C,D), suggesting transition from pro-inflammatory toward pro-resolution status, potentially preventing progression to fibrotic remodeling.

### Transcriptomic changes by exosomes treatment indicate restore disrupted cell–cell communication networks in the septic lung

**Figure 7.**
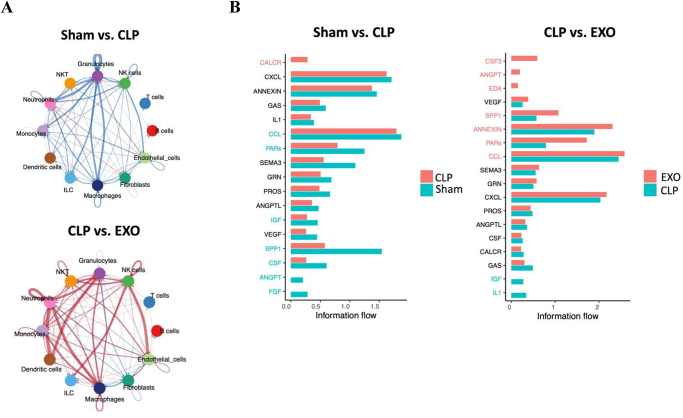
Cell–cell communication network analysis in lung tissue. (A) Network visualization of cell-cell interaction patterns for Sham vs. CLP (upper) and CLP vs. EXO (lower) comparisons. Red indicates upregulated interactions; blue indicates downregulated interactions. Note decreased cellular communication in CLP vs. Sham and its restoration following exosome treatment. (B) Ligand-receptor communication probability analysis for Sham vs. CLP (left) and CLP vs. EXO (right) comparisons. In CLP vs. EXO, communication probabilities of CSF3, ANGPT, SPP1, PARs, and CCL pathways show significant increases, while IL-1 and IGF pathways decrease, demonstrating rebalancing of intercellular signaling networks after exosome treatment.

To examine the intercellular interactions involved in sepsis-induced lung injury and assess the regulatory effects of ADSC-derived exosomes, we constructed a cellular communication network encompassing various cell types. Compared to the sham group, the interaction strength between cell types decreased overall in the CLP group but then increased following ADSC-derived exosomes treatment (Fig. 7A).

**Figure 8.**
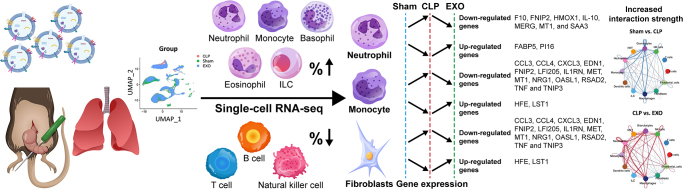
Adipose-derived stem cell exosomes protect against sepsis-induced lung injury through multiple coordinated mechanisms. ADSC exosomes significantly reduce lung pathology by altering cellular composition, increasing granulocytes, monocytes, and immature lymphoid cells (ILC), while decreasing natural killer cells, T and B cells. At the molecular level, exosomes downregulate pro-inflammatory genes across multiple cell types while enhancing tissue repair pathways. In neutrophils, exosomes reduce respiratory burst gene expression while promoting tissue regeneration mechanisms. In monocytes, they suppress inflammatory cytokine production while fostering anti-inflammatory phenotypes. Exosome treatment is associated with transcriptomic changes suggestive of restored intercellular communication networks and pathways disrupted by sepsis. Through these multifaceted actions, ADSC exosomes effectively rebalance the lung’s cellular environment, shifting the immune response from destructive inflammation toward healing and homeostasis.

Furthermore, we identified the ligand–receptor pairs. Compared to the sham group, the communication probabilities generally decreased in the CLP group, with the exception of CALCR (Fig. 7B). Following treatment with ADSC-derived exosomes, the communication probabilities of IL-1 and IGF decreased. The communication probabilities of CSF3, ANGPT, SPP1, PARs, and CCL, which were significantly reduced in the CLP group, increased significantly in EXO group (Fig. 7B). These findings indicate that ADSC-derived exosome treatment effectively alleviated the imbalance in the intercellular network caused by sepsis. Summary of the main findings of this study regarding how exosomes are protective against lung-related injuries in sepsis is illustrated in Figure 8.

## Discussion

ADSC-derived exosomes show remarkable potential in mitigating sepsis-induced ALI in mice, offering significant clinical relevance. With ALI/ARDS being a major cause of ICU mortality and lacking targeted treatments beyond supportive care^[29]^, these findings on improved survival and lung pathology are highly significant. Exosome therapy presents advantages over stem cell transplantation – they are less immunogenic, non-tumorigenic, and can be stored for on-demand use^[29]^. This suggests the possibility of an off-the-shelf therapeutic for septic patients at risk of ALI. By targeting immune dysfunction rather than just infection, ADSC exosomes align with the growing consensus on improving sepsis outcomes^[30]^.

In the comparison with recent studies, our results align with recent preclinical research showing MSC/ADSC exosome benefits in sepsis and ALI models. Wang et al. demonstrated ADSC-exosomes reduced macrophage aggregation in septic lungs, suppressed inflammatory cytokines, preserved vascular integrity, and improved survival in CLP models by inhibiting IL-27 secretion from macrophages^[20]^. Another study showed MSC-derived exosomes curbed neutrophil extracellular trap formation in septic lungs, reducing ALI severity^[31]^. Beyond protein signaling, exosomal non-coding RNAs serve as therapeutic mediators. Shen et al. identified circular RNA (circ-Fryl) in ADSC exosomes that activates pro-survival pathways in injured lungs, enhancing autophagy via the miR-490-3p/SIRT3 axis to suppress alveolar cell apoptosis and inflammatory cytokine production^[19]^. Xia et al. reported ADSC exosomes transfer functional mitochondria to alveolar macrophages, restoring bioenergetics and driving macrophages toward an anti-inflammatory phenotype^[18]^. Whereas the above studies each pinpoint a single pathway or cell type, our analysis reveals that exosomes act on multiple cell populations simultaneously. We observed coordinated transcriptional changes across neutrophils, monocytes, macrophages, lymphocytes, endothelial cells, and fibroblasts. Notably, exosome treatment downregulated numerous pro-inflammatory genes elevated by sepsis (e.g., *Tnf, Ccl3, Ccl4*, and even the immunomodulatory cytokine *Il10*) while upregulating reparative and pro-resolving factors such as *Cd93, Mmp9*, and *Egfl7* in the injured lungs. Although our results did not reveal any analogous IL-17 signal pathway or circRNA-mediated autophagy pathways, these broad gene expression shifts suggest that ADSC exosomes do not simply inhibit one cytokine (as with IL-27 in Wang et al.) or activate one cytoprotective pathway (autophagy or metabolism as in Shen et al. and Xia et al.), but rather trigger a network of anti-inflammatory and regenerative programs across cell types.

Our single-cell RNA-seq analysis revealed how exosome therapy recalibrates the lung’s cellular environment during sepsis. In untreated CLP mice, we observed expanded neutrophils and inflammatory monocytes with inflammatory transcriptional profiles, alongside dysregulated lymphocytes, endothelial and fibroblastic cells^[30]^. ADSC-exosomes reversed many of these changes: 1. neutrophils showed normalized expression of activation and respiratory burst genes, suggesting reduced tissue damage; 2. monocytes/macrophages shifted from hyperinflammatory M1-like states toward regulated M2-like states, with upregulated anti-inflammatory genes (e.g., Il10, Arg1) and downregulated proinflammatory cytokines; 3. lung fibroblasts, which acquired an activated phenotype in untreated CLP mice, showed tempered responses with exosome treatment, potentially preventing progression from acute injury to chronic fibrotic remodeling. The net effect is a restoration of homeostasis: dampened proinflammatory circuits, enhanced regulatory programs, and balanced cell populations.

In this study, our single-cell transcriptomic findings for neutrophils and monocytes in septic lung tissue show both concordance and divergence with recent studies in sepsis. Consistent with clinical data, we observed an expansion of an immunosuppressive monocyte subset in septic mice. Human sepsis studies have identified a CD14^+^ monocyte population with low HLA-DR and high S100A8/A9 expression that emerges during sepsis and is associated with immune paralysis^[32]^. Similarly, our septic mice in the absence of exosome treatment showed monocytes upregulating S100a8/S100a9, indicating a comparable immunosuppressive phenotype. However, monocytes in exosome-treated mice did not exhibit the pronounced HLA-DR suppression seen in human sepsis cohorts^[33]^. This suggests that the exosome therapy may preserve or restore antigen-presenting capacity in monocytes, whereas in untreated sepsis patients monocyte HLA-DR is often pathologically low^[32]^. One possible explanation is that MSC-exosomes convey immunomodulatory signals that prevent monocyte dysfunction, a divergence from the typical clinical pattern of sustained monocyte deactivation.

In addition, recent animal studies also highlight neutrophil heterogeneity in sepsis, which aligns with our observations. In severe sepsis, neutrophils can acquire an immature, low-density phenotype with high expression of inhibitory checkpoints like PD-L1^[34]^. Indeed, a 2025 study in septic mice showed that non-survivors had increased CXCR4^+^PD-L1^+^ neutrophils that suppress lymphocytes^[35]^. Our untreated septic group exhibited gene signatures consistent with highly activated neutrophils, including those associated with degranulation and oxidative bursts, which is in line with reports of hyperactivated neutrophil states in early sepsis^[34]^. In contrast, neutrophils from exosome-treated mice showed a tempered activation profile. We noted, for example, reduced expression of genes encoding proteases and ROS generators (trends toward a less damaging phenotype), alongside lower expression of PD-L1 compared to controls. This contrasts with the aforementioned findings in which PD-L1^+^ neutrophil subsets expand in more severe or unmitigated sepsis^[35]^. One potential reason for this divergence is that the exosome therapy might be blunting the “emergency granulopoiesis” and aberrant activation that drive neutrophil-mediated immunosuppression. It has been shown that controlling excessive neutrophil recruitment and differentiation can prevent progression to an “extreme immunosuppressive” endotype of sepsis^[36]^. Kwok and colleagues, for example, described an extreme endotype characterized by overwhelming neutrophil-driven inflammation alongside immune suppression^[36]^. The exosome-treated mice in our study may represent a shift away from that endotype – their neutrophil transcriptomes indicate reduced intensity of both inflammation and immunosuppressive signaling. Differences between our results and other studies may be attributed to the intervention and timing: our samples were taken at an acute time-point after exosome treatment, whereas many human studies examine later, unmodulated sepsis. Collectively, our findings agree with recent literature that sepsis triggers distinct immunoregulatory monocyte and neutrophil subsets^[34]^. The key similarity is the presence of immunosuppressive phenotypes (HLA-DR^low^ monocytes, PD-L1^high^ neutrophils) in sepsis^[34]^, while a notable divergence is that our therapeutic intervention appears to partially reverse these changes. This underscores a potential mechanism whereby MSC-derived exosomes modulate myeloid cell states toward a balance of controlled inflammation rather than unchecked paralysis or hyperinflammation.

Exosome treatment is associated with transcriptomic changes suggestive of restored cell–cell communication networks disrupted by sepsis. Through ligand–receptor interaction analysis, we observed that CLP caused breakdown of signaling axes between stromal and immune cells. Exosome administration enhanced intercellular communication by promoting anti-inflammatory signals and attenuating proinflammatory loops. This system-level realignment suggests exosome therapy does not target just one cell type or pathway but simultaneously modifies multiple nodes of the lung’s immune network.

Gene ontology and KEGG pathway enrichment provided mechanistic insights. In CLP lungs, neutrophils and monocytes showed enrichment in NF-κB signaling, cytokine interactions, and leukocyte chemotaxis pathways, while fibroblasts showed enrichment in extracellular matrix organization and TGF-β signaling. Exosome treatment reversed many of these enrichments, downregulating inflammatory pathways while upregulating cellular stress resistance, metabolic homeostasis, and repair pathways. We observed enrichment of oxidative phosphorylation and mitochondrial function in immune cells after exosome therapy, consistent with reports that ADSC exosomes enhance mitochondrial health in alveolar macrophages^[29]^. Increased autophagy and anti-apoptotic signaling were also evident, supporting the circRNA-mediated autophagy mechanism^[19]^. These pathway changes confirm exosomes act as master regulators, shifting the response from destructive inflammation toward healing.

ADSC-derived exosomes show promise for clinical evaluation, having demonstrated improved lung function without overt immunogenicity or toxicity^[29]^. Challenges remain before clinical adoption, including large-scale production standardization, ensuring exosomal cargo stability, and establishing optimal dosing^[37]^. By reestablishing immune equilibrium in the lung, ADSC-derived exosomes address the core of sepsis pathology, potentially emerging as a valuable treatment against sepsis and its pulmonary complications.

## Limitations and strengths

This strictly murine study, which employed CLP model of polymicrobial sepsis to induce ALI, provides key insights but also has inherent limitations in its experimental design. The CLP model is a commonly used, clinically relevant rodent sepsis model, but it cannot fully replicate the immunological complexity and protracted course of human sepsis. Moreover, we acknowledge that our single-cell RNA-seq analysis was conducted with only two biological replicates per group, which is a clear limitation. This small sample size reduces statistical power and makes it difficult to distinguish true biological signals from noise. As a result, some transcriptomic changes we report may reflect individual variability rather than broadly generalizable sepsis mechanisms. The limited replication also constrains our ability to capture the full heterogeneity of immune responses in sepsis. Therefore, we have tempered our conclusions and emphasize that these results are exploratory. In future studies, we plan to increase the number of animals per group to strengthen the robustness and generalizability of the single-cell findings. We also tested only a single dose and time-point of ADSC-derived exosome administration optimized for the acute phase; this dosing regimen, while practical, precluded evaluation of dose–response relationships or longer-term outcomes. Furthermore, the tissue scope of our analysis was confined to the lungs, providing depth in pulmonary pathology but not addressing potential exosome effects on other sepsis-vulnerable organs such as the kidney or liver. In addition, the absence of a sham exosome control in this study limits the interpretation of the results, as the influence of injection or vehicle-related effects cannot be disregarded. In future work, administering PBS or heat-inactivated exosomes to a septic group will help distinguish specific exosome-mediated effects from any nonspecific effects of treatment administration, thereby strengthening the rigor of our conclusions. The exclusive use of male mice and lack of proteomic validation also impose certain limitations on this investigation. This is an important limitation because immune responses in sepsis can differ between males and females. At last, the result of the histopathological change of lungs is judged by different assistants who were not blind to the mice’s condition. The absence of blinding means there is a risk of observer bias – knowledge of treatment groups could have inadvertently influenced the scoring of lung injury severity. Although we used predefined scoring criteria to guide evaluations, the potential for conscious or unconscious bias remains a limitation. We recognize that this could affect the objectivity of our histopathology results, and we will implement blinded evaluation protocols in future experiments to ensure more unbiased outcome assessments. In future work, the functional studies will provide causal evidence to support the correlative transcriptomic findings and help translate our results into therapeutic strategies. For instance, if we find that a specific inflammatory process, like NF-κB signaling or NLRP3 inflammasome activation, occurs in monocytes, then checking how active this process is (like measuring cytokine production or signaling protein changes) in cells with and without exosome exposure would be helpful. Likewise, top differentially expressed genes related to chemotaxis or oxidative burst identified in neutrophils will be examined using targeted knockdown or pharmacologic inhibition to see if changing their expression affects disease outcomes. Despite these constraints, the experimental design had notable strengths. We combined high-resolution scRNA-seq with survival analysis, connecting whole-animal results to cellular and molecular data. The scRNA-seq, in particular, enabled identification of distinct immune cell subsets and transcriptional changes with fine granularity that would be obscured in bulk assays. This multifaceted approach, combining phenotypic and molecular endpoints, strengthens the rigor and comprehensiveness of our findings.

Some translational barriers based on the study results also should be acknowledged. First, there are important immune differences between murine models and human sepsis. Mice are inbred and have a more synchronized immune response, whereas human sepsis patients are highly heterogeneous with diverse genetic backgrounds and comorbidities. Certain immune cell proportions and responses differ, meaning therapies successful in mice may not readily replicate in humans^[38]^. Second, producing clinical-grade exosomes in large quantities with reproducible properties is technically demanding^[39]^. Current isolation methods are low-throughput and can introduce variability. At last, rigorous validation in more human-like models is needed. Large animal studies will be instrumental to bridge the gap between mice and humans.

## Conclusion

Our study demonstrates that ADSC exosome therapy can alter immune-cell gene expression profiles and ameliorate lung injury in a preclinical sepsis model. We stress that these findings are preliminary and specific to our mouse experiments. While the data reveal potential therapeutic mechanisms – such as reprogramming of neutrophils and monocytes toward less pro-inflammatory states – we cannot claim any definitive clinical efficacy. The results support the concept that MSC-derived exosomes may modulate the septic inflammatory response, but further validation is required. We now explicitly note that this is an early-stage investigation and that extensive additional research, including functional experiments and trials in larger animals and humans, will be needed to confirm the translatability of these benefits. Ultimately, our revised conclusion emphasizes that the exosomes show promise as a novel therapy to explore, but their effectiveness and safety must be proven in future studies before any clinical application is considered.

## Data Availability

Data sharing not applicable – no new data generated.

## Announcement of AI assistance

The use of AI for manuscript drafting is consistent with the TITAN 2025 standards^[40]^, which require complete transparency in AI interaction. OpenAI GPT-4o was used for reference searching, with inputs limited to publicly available literature metadata from Pubmed, Scopus, and Medline. No statistical analysis or image production was done. No plugins or local deployments were used. OpenAI GPT-4o was also used to check for spelling, grammar, syntax, and reference correction. The complete prompt transcripts for chatGPT were: Determine whether there is an abbreviation without a full name before it, or whether the full name is repeated in the text despite the fact that an abbreviation has previously been provided. Then, as a reviewer, examine for faults in the linked reference’s textual description. Examine the consistency of in-text citations and metadata (e.g., author, title, journal), as well as whether the research descriptions match the cited articles. Report any minor formatting, grammar, syntax, or citation issues. All AI-generated text was fact-checked, evaluated, or revised by the senior author; non-verifiable results were discarded. The authors declare no financial relationships to AI suppliers and adhere to the International Committee of Medical Journal Editors and World Medical Association ethical guidelines.
